# Endoscopic multispectral imaging sensor systems for intraoperative tissue differentiation

**DOI:** 10.1117/1.JBO.31.9.097003

**Published:** 2026-09-22

**Authors:** Andrea Rüdinger, Kira Holstein, Alissa Tarnoruzki, Nora Lösch, Tobias Haist, Stephan Reichelt

**Affiliations:** University of Stuttgart, Institute of Applied Optics (ITO), Stuttgart, Germany

**Keywords:** multispectral imaging, endoscopic imaging, cancer, tissue differentiation, hyperspectral imaging

## Abstract

**Significance:**

To remove the entire tumor area in cancer surgery, the margins are currently assessed by frozen sectioning or by analyzing the resected tissue afterwards. By utilizing spectral differences between healthy and malignant tissue, multispectral imaging (MSI) enables real-time tissue differentiation. Through miniaturization, the system can be used intraoperatively, thereby enabling the surgeon to define tumor margins, which reduces the need for a follow-up surgery.

**Aim:**

By obtaining characteristic spectral differences of healthy and malignant breast tissue, a miniaturized multispectral sensor system should be developed. The system should be compatible to commercial endoscopes, so that it can be used in minimally invasive endoscopic procedures.

**Approach:**

We analyzed hyperspectral measurements of an *ex vivo* breast cancer study in the visible to shortwave infrared spectral range (450 to 1390 nm) and defined characteristic spectral differences. With these, we designed two MSI sensor systems, which only use few spectral channels, gaining a potentially real-time-capable sensor system. By miniaturization, they can be used intraoperatively with a commercial endoscope.

**Results:**

The final setups can be used with a commercial endoscope and allow for 90 deg field of view and a high spatial resolution, but showed limitations in the SWIR transmission.

**Conclusion:**

The two demonstrated systems are promising steps towards real-time endoscopic tissue differentiation.

## 1 Introduction

As minimally invasive procedures become increasingly common, the need for intraoperative imaging and sensor technology is growing to enable surgeons to analyze tissue despite reduced haptic feedback and the lack of direct visual assessment. To support the surgeon in analyzing the perfusion of distinct regions or differentiating tissue areas, several techniques have recently been developed and are commonly used. These include fluorescence imaging, in which fluorescence markers such as indocyanine green (ICG) are dispensed and for example, adsorbed in lymph nodes, enabling the detection of the sentinal lymph node in breast cancer surgery for evaluation of the axillary lymph node status.^1^ Along with lymph node detection,^1^^–^^3^ ICG is also used for perfusion monitoring^3^^–^^6^ and tumor detection.^7^^–^^9^ However, fluorescence imaging deals with several limitations, such as limited observation time.^3^^,^^4^

Another common method is narrow-band imaging (NBI), which uses two to three narrow spectral channels in the visible (VIS) wavelength range to illuminate the tissue surface. By the different penetration depths, tissue structures such as capillaries and blood vessels are contrast enhanced to the surrounding mucosa. Therefore superficial mucosal lesions can be better detected and analyzed.^10^^–^^12^

By analyzing the reflected light, hyperspectral imaging (HSI) allows for intraoperative perfusion monitoring, tissue differentiation and image guided surgery^13^^–^^17^. In the last years, several setups, from laboratory prototypes^18^^–^^22^ to commercially available systems^23^^,^^24^ (Tivita Tissue, Diaspective Vision) have been developed, either using only the visible to near infrared (NIR) and shortwave infrared (SWIR) wavelength range or both.

To improve the often high acquisition time of HSI systems, using only a few spectral channels is a possible solution. This not only reduces data acquisition time but also minimizes the time and resources required for data processing. In case of perfusion monitoring, such multispectral imaging (MSI) systems have already been used in the VIS-NIR spectral range,^20^^,^^25^^,^^26^ for example, by either using a multispectral snapshot camera^27^ or multispectral illumination of the tissue by distinct spectral channels.^28^ Fischer et al.^29^^,^^30^ used this principle for tissue differentiation in case of healthy and malignant bladder tissue, by selecting just three narrow spectral bands.

In this context, we want to present two multispectral sensor systems for endoscopic tissue differentiation. These allow for marker-free, potentially real-time capable data acquisition and processing, reduced computational resources and for integrability to standard endoscopes. Therefore, we introduce the procedure of defining meaningful spectral channels and present the implementation in Sec. 2. Section 3 shows characterization results of both sensor modalities.

## 2 Materials and Methods

### 2.1 Data Analysis

#### 2.1.1 Patient study

To define characteristic spectral channels for tissue differentiation, a hyperspectral dataset of breast cancer has been used. This dataset arised from an *ex vivo* patient study taking place at the Tübingen University Hospital from November 2024 to July 2025. The study was in accordance with “The Code of Ethics of the World Medical Association” (Declaration of Helsinki) and approved by the Ethics Committee of the University of Tübingen (UKT IRB# 440/2023/BO2). In total, 46 patients were enrolled in the tissue differentiation study of the Research Training Group (RTG) 2543 after providing informed consent. The patients were diagnosed with ductual carcinoma in situ (DCIS) or cT1, cT2, cT3, and cT4 breast cancer. Of these 46 patients, 38 patients aged between 37 and 89 (36 female and 2 male, average age: 59.5 years) could be used for the further spectral analysis. Most of the patients had received treatment before surgery. Two patients were treated with neoadjuvant chemotherapy, two patients received neoadjuvant chemotherapy in combination with immunotherapy, two patients were treated with neoadjuvant chemotherapy in combination with an antibody-drug-conjugate, one patient received a neoadjuvant chemotherapy in combination with antiendocrine therapy, and 20 patients were treated with an antiendocrine therapy before surgery.

The measurements were embedded into the clinical routine as described in Rüdinger et al.^31^^–^^33^ Patients were measured by the monochromatically scanning hyperspectral measurement system presented by Rüdinger et al. in Refs. 31, 33, 34. The measurement system consists of a custom monochromator, which splits the broadband light of a halogen light source (Thorlabs OSL2IR) into 101 narrow spectral bands (${\overset{¯}{\mathrm{FWHM}}}_{\mathrm{VIS}}=12.53\;\mathrm{nm}\pm 0.59\;\mathrm{nm}$ for 450 to 950 nm and ${\overset{¯}{\mathrm{FWHM}}}_{\mathrm{SWIR}}=9.1\;\mathrm{nm}\pm 2.93\;\mathrm{nm}$ for 950 to 1450 nm, used spectral range for this study: 450 to 1390 nm). The light is then coupled into a fan-out fiber bundle (Thorlabs BFL44LS01, diameter single fiber core: 400  μm, vertical orientation), which distal ends are placed around the objective lens of the imaging part of the system to illuminate the tissue surface. The reflected light is separated by a dichroic mirror (Thorlabs DMSP950T) and imaged on two image sensors (VIS/NIR: Ximea MQ013 MG-E2, objective: Edmund Optics #67-714; SWIR: Raptor Photonics Ninox 640 SU, objective: Computar ViSWIR Lite M1614-VSW). In total, an object field of 15  mm×15  mm with a spatial resolution of 22  μm for VIS/NIR and 56  μm for SWIR can be measured.

#### 2.1.2 Data preprocessing

For preprocessing, the hyperspectral cube is first corrected for the individual exposure times per spectral channel. Afterwards, a correction regarding internal reflections as well as the camera sensors sensitivity and the inhomogeneity of the illumination is performed [see Eq. (1)]. For this purpose, a dark and a white image are measured using a highly absorbing foil (Edmund Optics, #12-695 Acktar Metal Velvet Foil) and a spectralon (Sphere Optics, Zenith Polymer Reflectance Standard).

$$I_{\mathrm{obj},\mathrm{sens},\mathrm{corr}}=\frac{\frac{I_{\mathrm{obj},\text{raw}}(\lambda )}{t_{\mathrm{obj},\exp}(\lambda )}-\frac{I_{\text{dark},\text{raw}}(\lambda )}{t_{\text{dark},\exp}(\lambda )}}{\frac{I_{\text{white},\text{raw}}(\lambda )}{t_{\text{white},\exp}(\lambda )}-\frac{I_{\text{dark},\text{raw}}(\lambda )}{t_{\text{dark},\exp}(\lambda )}}.$$ (1)

The two hyperspectral cubes are combined by aligning the camera coordinate systems of the VIS/NIR and SWIR camera (alignment error≤5  pix). A min-max normalization is performed along the spectral axis of the combined hypercube, to enable spatial comparison of the spectra and to consider intensity variations due to surface topology.^31^^,^^33^

#### 2.1.3 Defining spectral channels

To analyze spectral differences of the different tissue types, the measurement images are registered manually with histological ground truth data by the specified scale of the hematoxylin and eosin stained (HE) sections and the tissues’ morphology. Because the tissue might be distorted due to the embedding process and the HE sections are extracted from deeper cell layers than the HSI measurements, the registration is challenging. To be conservative, especially in the margins of the tumor area, only regions of interest (ROI), in which a clear pathological label can be defined, are selected from the measurement images.^31^^–^^33^

**Fig. 1:**
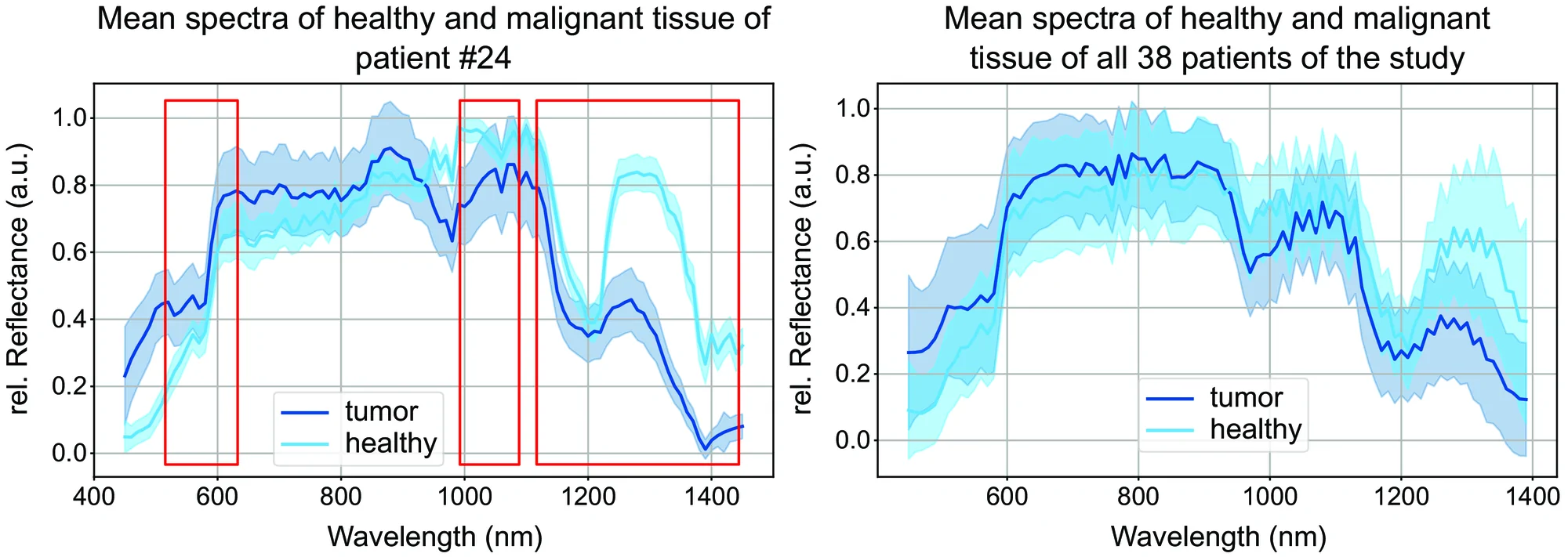
Mean spectra of healthy and malignant tissue based on the ROIs of patient #24 (left) and for all 38 patients of this study (right). Shaded areas represent the standard deviation of the single spectra within the ROIs. Red boxes highlight the regions of spectral differences.

As mentioned in Rüdinger et al.,^31^^–^^33^ there are spectral differences, which allow for differentiation between healthy and malignant tissue. These spectral differences are not only visible for an individual patient but can also be seen in the mean tumor and healthy tissue spectra of all patients of the study, as shown in Fig. 1. The highlighted regions not only differ in the absolute relative reflection but also in the gradient and curvature of the spectra.

**Fig. 2:**
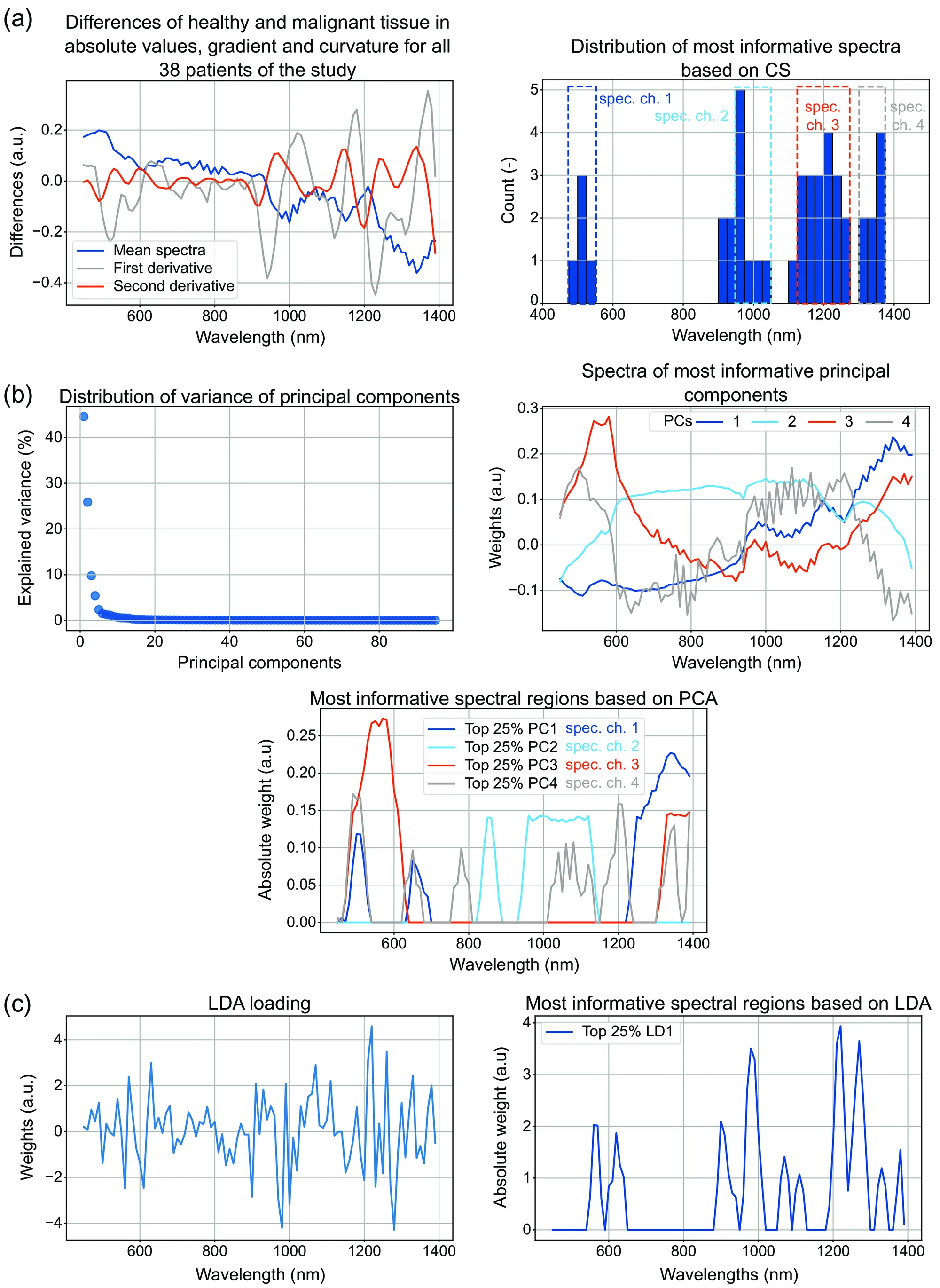
Spectral reduction based on curve sketching (a), PCA (b), and LDA (c). The most informative spectral ranges are obtained by selecting the top 25% of the absolute values of the first and second derivative of CS, the PCs, or the LD. As can be seen, for all three methods similar spectral regions are selected. Spectral channels used for further classification, either by CS or PCA, are highlighted.

For further analysis, the spectra of the tumor and healthy tissue is analyzed by a curve sketching method (CS) presented in Rüdinger et al.,^33^ by Principal Component Analysis (PCA) and by Linear Discriminant Analysis (LDA), see Fig. 2. The most informative spectral ranges are selected by taking only the top 25% of the absolute values of the first and second derivative in case of CS, the PCs or the LD. For better comparison, the most important wavelengths of each method are listed in Table 1. It can be seen that all of the methods show similar results and therefore confirm the importance of these spectral ranges.

**Table 1:** Most important spectral channels for differentiation of healthy and malignant tissue based on curve sketching (CS), principal component analysis (PCA), and linear discriminant analysis (LDA). All methods show comparable spectral ranges, which confirms their importance.

CS (nm)	PCA				LDA (nm)
	PC1 (nm)	PC2 (nm)	PC3 (nm)	PC4 (nm)	
480 to 550	460 to 530	830 to 880	450 to 630	450 to 530	560 to 630
970 to 1050	640 to 690	940 to 1140	1310 to 1390	630 to 670	900 to 930
1130 to 1250	1230 to 1390			760 to 800	970 to 1000
1300 to 1390				1020 to 1130	1070 to 1100
				1160 to 1230	1200 to 1300
				1310 to 1390	1380

**Fig. 3:**
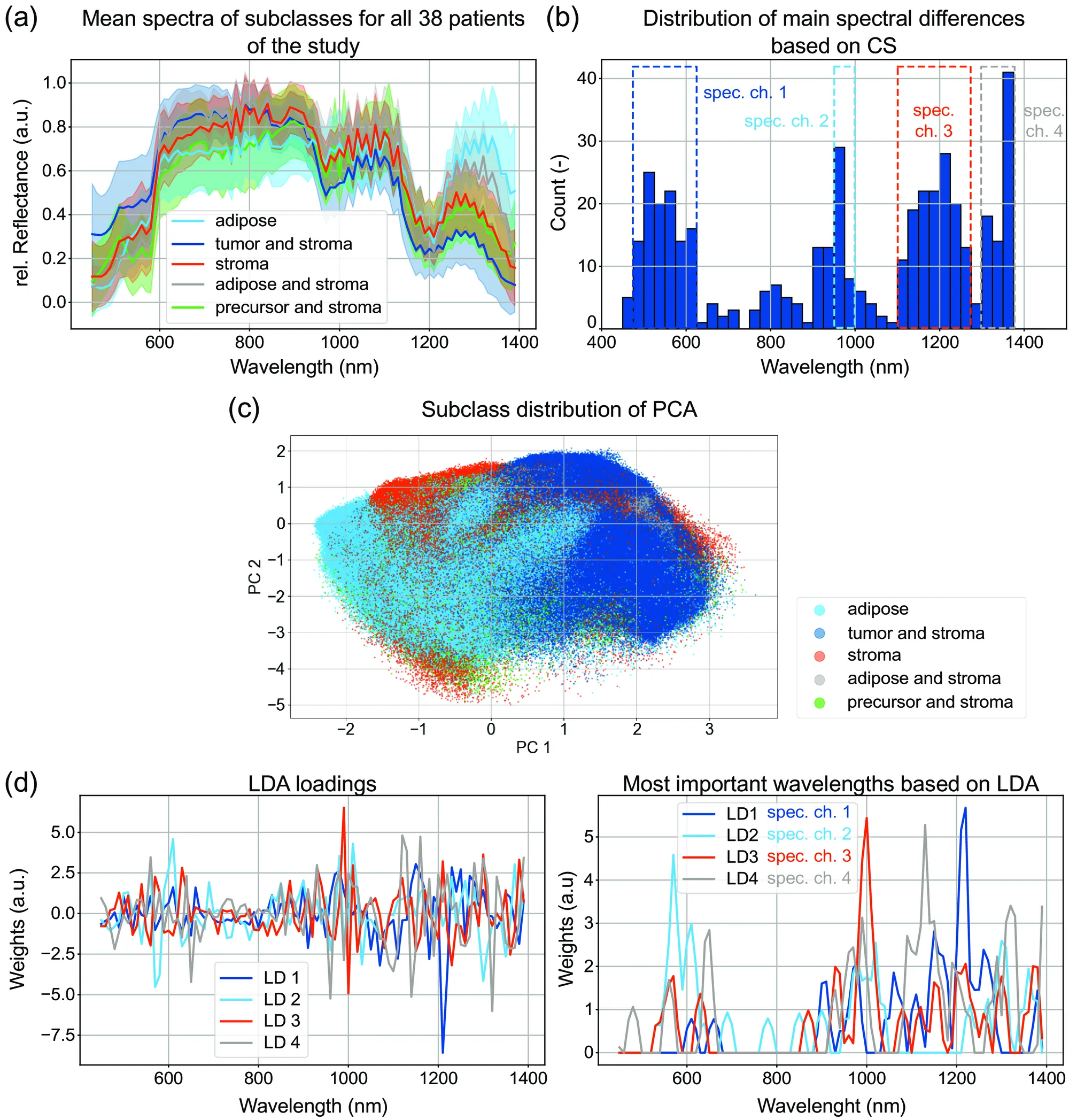
Spectral reduction of multiclass problem. (a) Mean spectra of the tissue subclasses and (b) spectral reduction based on curve sketching. Because the PCA is an unsupervised learning algorithm, the result of the PCA is the same as for healthy and malignat tissue, shown above. The resulting clustering of the PCA is shown in panel (c). For LDA, four LDs are obtained and shown in (d). As shown, the most informative spectral ranges are obtained by selecting the top 25% of the absolute values of the first and second derivative of CS, the PCs or the LDs. Spectral channels used for further classification, either by CS or LDA, are highlighted.

Because the dataset is biased by more elderly patients, the amount of adipose tissue in the healthy tissue class is increased. To be aware of this, the class labels are adapted to ‘tumor and stroma’, ‘stroma’, ‘adipose and stroma’, ‘adipose’, ‘precursor and stroma,’ and ‘undefined’ (in case of obstacles or background). The results of mean spectra, curve sketching, PCA, and LDA are shown in Fig. 3. Table 2 shows the most important spectral channels. It can be seen that the results are comparable to the two-class evaluations and only minor differences in the spectral channels selected can be seen.

**Table 2:** Most important spectral channels for differentiation of subclasses of healthy and malignant tissue based on curve sketching (CS), principal component analysis (PCA), and linear discriminant analysis (LDA). All methods show comparable spectral ranges, which confirms their importance.

CS (nm)	PCA				LDA			
	PC1 (nm)	PC2 (nm)	PC3 (nm)	PC4 (nm)	LD1 (nm)	LD2 (nm)	LD3 (nm)	LD4 (nm)
480 to 630	460 to 530	830 to 880	450 to 630	450 to 530	610	540 to 630	540 to 630	480
970 to 1000	640 to 690	940 to 1140	1310 to 1390	630 to 670	660	690	870	560
1100 to 1280	1230 to 1390	—	—	760 to 800	900 to 980	780	930 to 1020	640 to 650
1300 to 1380	—	—	—	1020 to 1130	1050 to 1070	850	1080 to 1260	930 to 1000
—	—	—	—	1160 to 1230	1110 to 1180	900	1300 to 1380	1090 to 1220
—	—	—	—	1310 to 1390	1210 to 1280	960 to 1030		1280 to 1330
—	—	—	—	—	1380	1230 to 1370	—	1390

**Fig. 4:**
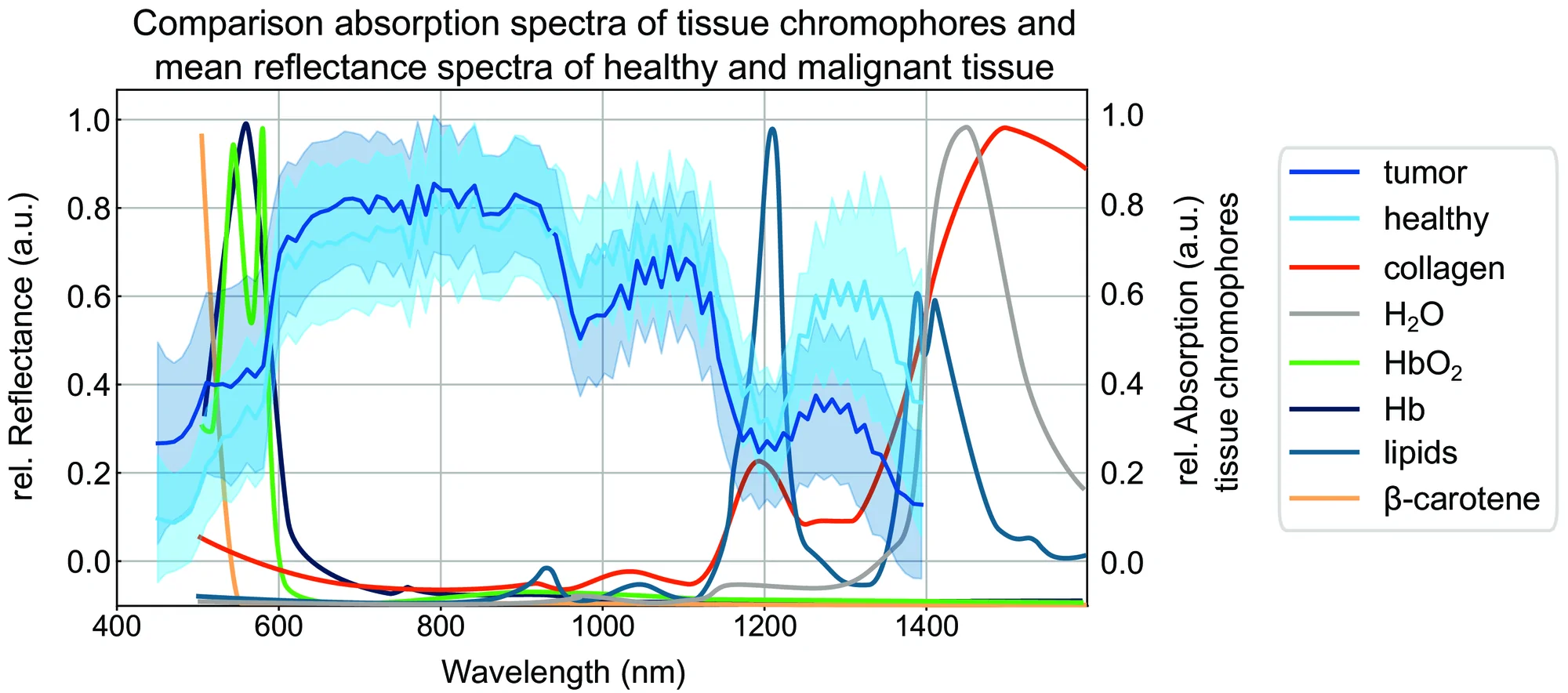
Comparison of mean spectra of healthy and malignant tissue and tissue chromophores of Nachabé.^35^ Main differences can be attributed to lipids and β-carotene for healthy and collagen and water for malignant tissue.

These spectral differences could be assigned to differences in the tissue components, such as lipids and β-carotene for healthy and collagen and water content for malignant tissue, which can be seen by comparing the mean spectra with the absorption spectra of tissue components from Nachabé et al.^35^ in Fig. 4. Because the healthy tissue class is biased by adipose tissue, the presence of lipids and β-carotene (yellowish color of the adipose tissue) becomes apparent. These are also described by Bydlon et al.^36^ as chromophores for adipose tissue. The chromophores in tumors can be attributed the tumors’ influence on healthy tissue, as they create a tumor microenvironment, which can lead to increased levels of collagen and water.^37^^–^^39^ It can therefore be assumed that the detection of tumor tissue is primarily achieved by the tumors microenvironment.

### 2.2 Classification

To proof the hypothesis that less spectral channels can still achieve a sufficient tissue differentiation, classification is done for full spectral and for different sets of reduced spectral information. For this purpose, the classification algorithms are first trained and tested on the previous mentioned ROIs, in which a clear pathological label can be defined. Due to the variance of the spectra within the ROI, which represent 1  mm×1.3  mm to 10  mm×10  mm of the tissue surface and therefore varying tissue composition (local changes of the single main tissue type), no spatial cross-validation is performed. Furthermore, because the ROIs also contain noise pixel, as from specular reflections, the variance is further increased, which helps prevent overfitting during training. The pretrained classifiers are then tested on the entire measurement images to obtain pixelwise prediction maps.

For this, the preprocessed measurement images are scaled to reduce the computational effort. The spectra are averaged during scaling so that the pixel size in object space is reduced to ∼100  μm.^33^ The entire measurement images are labeled using semiautomated generated label masks, defined by the registration of the HE sections and the preprocessed measurement images as outlined before. Because of the mentioned uncertainties, there might be pixels, which are labeled with false labels and therefore lead to falsely classified pixels. Nevertheless this approach is more precise than the approach used in Rüdinger et al.,^33^ which uses only one label for the entire image, even if there is healthy tissue surrounding the tumor.

For classification a dense neural network (NN) and a Light Gradient Boosting Machine classifier (LightGBM) with default parameters are used. Both algorithms allow for fast training and prediction and resulted into comparably good or better results in previous classification runs as a Support Vector Machine or Random Forest classifier, which took longer computation time.

The input to both classifiers is the full spectrum of a single pixel of the ROI or of the entire image, given between 450 and 1390 nm with a stepsize of 10 nm, resulting in a vector of length 95. In case of reduced spectral information, the input shape is reduced to four spectral channels, either by CS, PCA, or LDA. The intensities of each spectral channel are obtained by integrating the tissue spectra (HSI measurement data) over the spectrum of the single spectral channel defined in Sec. 2.1.3, masking less relevant spectral regions. The spectra of the spectral channels are modeled as a binary function of amplitude one in case of informative ranges and zero else.

The NN consists of three hidden layers, with 64 nodes in the first, 32 nodes in the second, and 16 nodes in the third hidden layer. Rectified linear unit functions (ReLU) are used as activation functions for the hidden layers. The output layer consists of two nodes with a softmax activation function. The loss during training for 100 epochs is computed as categorical crossentropy. In case of label subclasses, the output shape is adapted to six.

The datasets (ROI and images) are splitted into a training and validation dataset. The split is performed patient-wise, with the aim of including as many patients as possible in the training because of the small sample size while ensuring a broad spectrum of diagnoses in both datasets. Further information is provided in Tables 3 and 4.

**Table 3:** Distribution of training and test dataset in case of tumor and healthy tissue classification.

	Training	Test	Total
Patients	31	7	38
Spectra absolute	4,688,519	673,927	5,362,446
Spectra relative	87%	13%	100%
Tumor	36%	66%	40%
Healthy	64%	34%	60%

**Table 4:** Distribution of training and test dataset in case of subclass classification.

	Training	Test	Total
Patients	31	7	38
Spectra absolute	4,688,519	673,927	5,362,446
Spectra relative	87%	13%	100%
Tumor and stroma	27%	52%	30%
Stroma	22%	25%	22%
Adipose and stroma	7%	2%	6%
Adipose	37%	18%	35%
Precursor and stroma	6%	2%	7%

The results of the classification runs with full and reduced spectral information are listed in Table 5 for tumor and healthy tissue and in Table 6 for subclasses.

**Table 5:** Classification results of the ROI and the entire image dataset for healthy and malignant tissue.

Dataset	Algorithm	Spectral channels	Accuracy (%)	Precision (%)	Recall (%)	F1-Score (%)
ROI	NN	95	81	83	81	81
ROI	NN	4 CS	78	81	78	79
ROI	NN	4 PCA	76	80	76	76
ROI	LightGBM	95	75	80	75	76
ROI	LightGBM	4 CS	79	82	79	79
ROI	LightGBM	4 PCA	76	80	76	77
Images	NN	95	85	88	85	86
Images	NN	4 CS	77	81	77	78
Images	NN	4 PCA	79	82	79	80
Images	LightGBM	95	86	87	86	87
Images	LightGBM	4 CS	77	81	77	78
Images	LightGBM	4 PCA	79	82	79	80

In case of main tissue classes [see Table 5 (top)], it can be seen that the classification results of reduced spectral information show comparably good results to the ones with full spectral information.

In a second step, the pretrained algorithms are tested on the measurement images. Pixels showing obstacles or background of the tissue sample are excluded because they were not used for training. The results of the main tissue type prediction of the entire image data (see Table 5 (bottom)), are comparable to the ROI results, which might be due to the scaling and therefore averaging the pixel spectra leading to reduced noise. Because the ROIs also contain noise spectra (e.g., from specular reflections) overfitting during training was prevented. One reason why the results are not better despite the lower noise level could be that the image spectra contain mixtures of different tissue types, especially in the margins. As light also penetrates the tissue, particularly the longer wavelengths, the spectra not only contain information from the tissues surface but also from deeper layers. However, it is to be expected that the signal strength from deeper layers will be lower than from the tissue surface. Nevertheless, this continues to result in signal mixing in the tissue spectra. In case of reduced spectral information, the classifiers ability to compensate for remaining noise and spectral signal mixing is reduced and thus leads in a less confident tissue type prediction in comparision to full spectral information, but still shows comparable results as for the ROIs.

The evaluations of the tissue type subclasses [see Table 6 (top)] show comparable results to the main classes in case of full spectral information, which are reduced by using less spectral information. This could be because the spectra of some subclasses show only minor differences that can no longer be clearly distinguished due to a shortage of spectral information. In addition, several tissue subclasses (’adipose and stroma’ and ’precursor and stroma’) only show minor portion in the distribution of the training dataset, because of the imbalance of the data, further impeding the prediction.

**Table 6:** Classification results of the ROI and entire image dataset for subclasses of healthy and malignant tissue. Weighted mean values are used as metric.

Dataset	Algorithm	Spectral channels	Accuracy (%)	Precision (%)	Recall (%)	F1-Score (%)
ROI	NN	95	81	80	81	80
ROI	NN	4 LDA	37	48	37	35
ROI	NN	4 CS	64	64	64	63
ROI	NN	4 PCA	67	71	67	68
ROI	LightGBM	95	76	76	76	75
ROI	LightGBM	4 LDA	50	59	50	50
ROI	LightGBM	4 CS	65	64	65	64
ROI	LightGBM	4 PCA	68	68	68	67
Images	NN	95	61	64	61	57
Images	NN	4 LDA	41	45	41	39
Images	NN	4 CS	48	47	48	45
Images	NN	4 PCA	49	48	49	47
Images	LightGBM	95	61	64	61	56
Images	LightGBM	4 LDA	41	46	41	39
Images	LightGBM	4 CS	48	47	48	44
Images	LightGBM	4 PCA	48	46	48	45

The reduction in results by reducing the spectral information is particularly true when predicting the overall images [see Table 6 (bottom)]. Especially in cases of less distinct tissue types, as precursor lesions, several aspects as less impact to the surrounding tissue, uncertainties in localization and registration, as well as superimposition with healthy tissue spectra leads to uncertainties in the tissue type prediction. High similarities between the tissue classes (‘adipose’ and ‘adipose and stroma’) also increase the complexity of the classification. Because the spectral information is reduced, the ability to compensate for uncertainties or only slight spectral differences between tissue subclasses is limited and may lead to ambiguities in tissue differentiation. Mixtures of different subclass spectra, as in case of the entire measurement images, further impede the classification and lead to poor classification results.

It is therefore shown that tissue classification in case of healthy and malignant tissue as well as for subclasses of healthy and malignant tissue with reduced spectral information is possible.

Because the number of patients participating in this study is still small, these factors could be minimized by conducting additional measurements, which would yield a more balanced dataset and, consequently, more robust spectral features, particularly for the subclasses. Furthermore, further optimization of the spectral channels used, either by increasing their number or the complexity of their spectra, is a conceivable option.

### 2.3 Variants of Multispectral Endoscopic Sensor Systems

To enable intraoperative tissue differentiation, we present two possible implementations of a multispectral sensor system, using the spectral channels presented in the previous sections. Both systems use a commercial endoscope (Karl Storz, Hopkins 30 deg, #26003BA) with light guide (Karl Storz, #495NB) and a VIS-SWIR image sensor (Basler ace 2 a2A640-240umSWIR).

#### 2.3.1 Illumination-based approach

The first implementation is an illumination-based approach. A customized light source is coupled into the commercial light guide, which is fixed to the illumination path of the endoscope.

For imaging, the imaging path of the endoscope is used. To allow for measurement of the NIR/SWIR spectral channels as well, the endoscope camera is changed to a VIS-SWIR image sensor (Basler ace 2 a2A640-240umSWIR, imaging lenses: Edmund Optics #49-659 and #49-926).

The light source is built by five LEDs (Thorlabs M565D2, M970D3, M1200D3, M1300D3, and M1450D3 with collimation lenses ACL12708U with optional A or B coating), covering the most important wavelength ranges obtained by CS and PCA.

The triggering of the LEDs is done by customized electronics, using an Arduino setup adapted from the OpenSFDI project.^40^ To combine the single LEDs, dichroic mirros (Thorlabs DMSP805 and DMSP1000) and right angle prism mirros (Thorlabs MRA15-P01 and MRA15-M01) are used. The prisms reflect the light of two different paths to be located excentrically on the focusing lens (focal length 60 mm) and than coupled into the light guide. The different angles of incidence to the light guide of the three light paths do not have an effect at the distal end of the endoscope because the light is mixed sufficiently in the light guide and the illumination path of the endoscope.

**Fig. 5:**
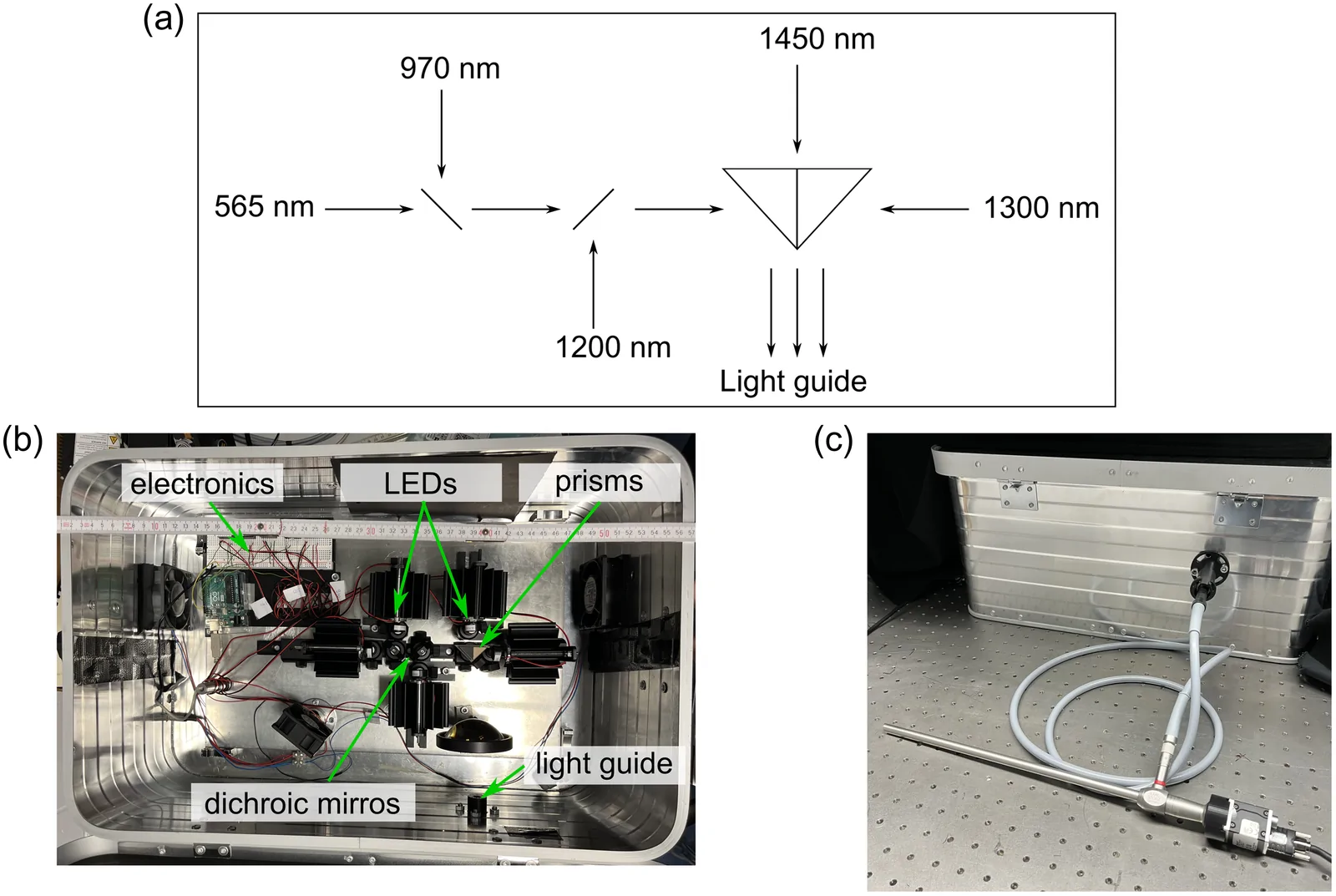
Schematic setup of the light source (a) and photo of the light source (b). Panel (c) shows the final endoscopic system.

By switching between the illumination modes, the multispectral cube can be acquired. Due to the modular design of the light source, the illumination can be realized by single LEDs or by combinations, to adapt to the spectral channels obtained by PCA. By adding additional LEDs, the spectra can be further adapted to better match the predefined spectra. Figure 5 shows the miniaturized measurement setup as well as the light source. The final dimensions of the imaging adapter are 38 mm in diameter and 60 mm in length (camera included) and has therefore comparable size to a standard endoscopic camera head. The light source is placed onto a 290  mm×450  mm aluminium board, which is placed into a larger box to allow for stray light shielding and a separate cooling system (dimensions width × length × height: 380  mm×590  mm×270  mm).

#### 2.3.2 Imaging-based approach

The second setup uses spectral filtering in the imaging path to implement the individual spectral channels. Unlike the illumination-based approach, this implementation is a snapshot method, allowing for capturing images of all four spectral channels at once. Because more complex spectral channels, as offered by the PCA, achieve higher classification results in case of subclass evaluation, this setups also uses complex spectral channels.

Due to limited mechanical space at the proximal end of the endoscope, a customized spectral filtering approach, based on various long- and short-pass filters, is not possible without extending the setup to a bulky device. On the contrary, designing customized multiband spectral filters is also complex with conventional dielectric filter design. This system is therefore based upon the polarization-based method, mentioned by Kudenov et al.^41^ and Hagen et al.^42^

**Fig. 6:**
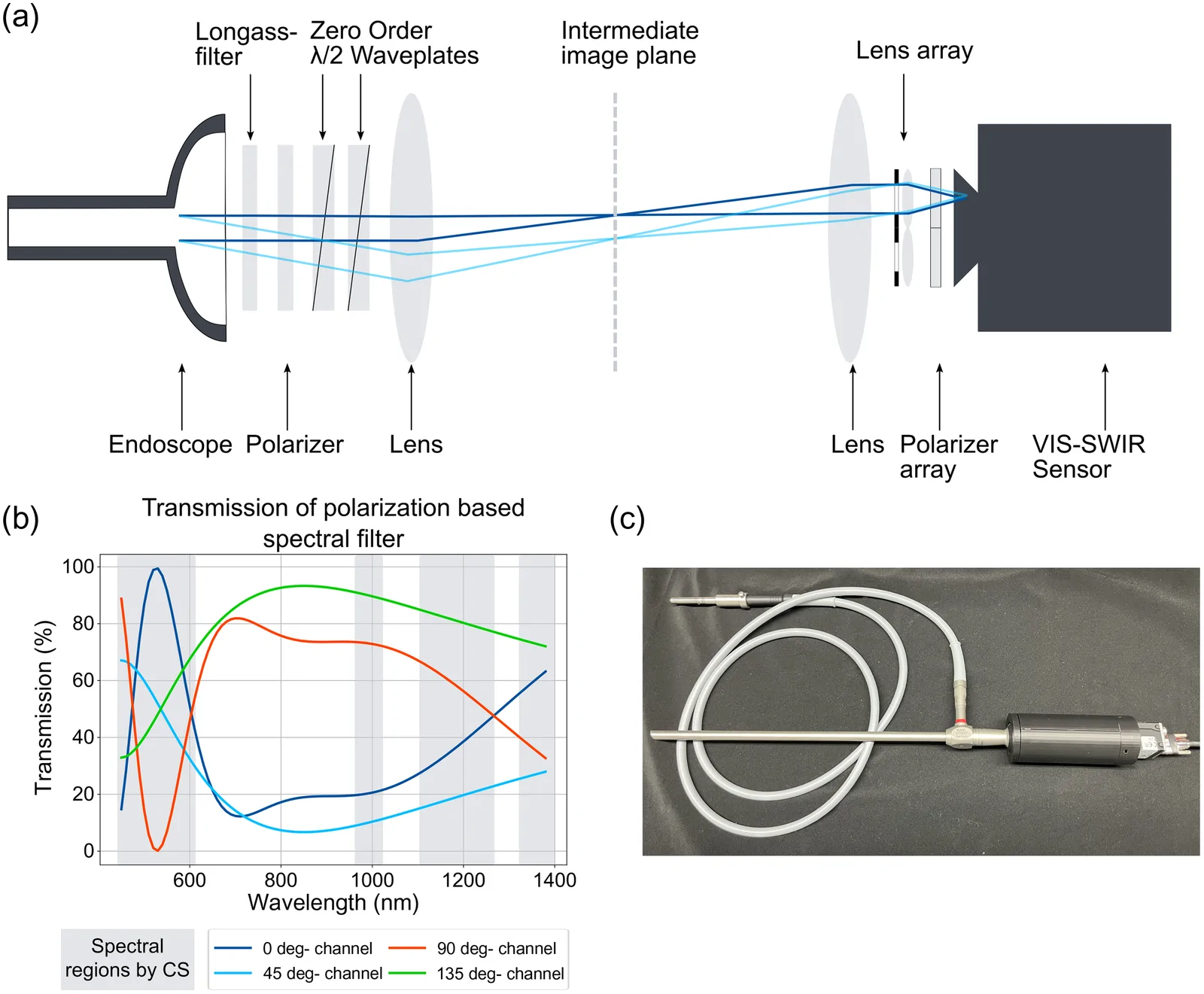
Schematic setup of the polarization-based sensor is shown in panel (a). Panel (b) shows the transmission spectra (spectral regions identified by CS are highlighted in gray for comparision) of the spectral channels. Panel (c) photo of the final endoscopic system.

Figure 6(a) shows the principle measurement setup. It consists of a linear polarization film (Edmund Optics #25-107), which is placed directly at the proximal end of the endoscope with an angle of $\alpha_{P1}=80\;\deg$. Then two zero-order quartz wave plates (Thorlabs WPH05M-850 and WPH05M-1550) are inserted with rotation angles of $\alpha_{\mathrm{WP}1}=10\;\deg$ and $\alpha_{\mathrm{WP}2}=45\;\deg$. To adapt the spectral range to the one measured and evaluated before, an additional long-pass filter is added (Edmund Optics #62-975). To expand the mechanical space, a relay lens system is used (two times Edmund Optics #49-335). After the intermediate image plane, the light becomes collimated again and than imaged to the camera sensor by a customized 2×2 lens array (4 times Edmund Optics #65-566). The lens array is combined with a customized polarization array, build by four polarizers (Edmund Optics #25-107) cut, and reassembled to the desired orientations. Finally, four images with $\alpha_{P2}=0\;\deg$, 45 deg, 90 deg and 135 deg are obtained, each representing a single spectral channel [Fig. 6(b))]. Figure 6(c) shows the final endoscopic measurement setup, which has 50 mm in diameter and 150 mm in length (camera included).

The thicknesses of the used wave plates, their rotation, and the rotation of the input polarizer are obtained by optimizing the accuracy of the classification task. For this purpose, the measured spectra of the ROI dataset are traced through a simulation of the final polarization-based MSI setup. This is done using the Jones formalism^43^^–^^45^, see Eq. (2).

$$J_{\text{out}}(\lambda )=J_{P2}\cdot J_{\mathrm{WP}2}(\lambda )\cdot J_{\mathrm{WP}1}(\lambda )\cdot J_{P1,\text{in}}.$$ (2)

To determine the Jones matrices of rotated wave plates $J_{\mathrm{WP},\mathrm{rot}}$, Eq. (3) can be used.^43^^,^^45^

$$J_{\mathrm{WP},\mathrm{rot}}(\alpha_{\mathrm{WP}})=J_{\mathrm{rot}}(-\alpha_{\mathrm{WP}})J_{\mathrm{WP}}J_{\mathrm{rot}}(\alpha_{\mathrm{WP}}).$$ (3)

Thereby $J_{\mathrm{rot}}$ is a rotation matrix, rotating the wave plate for a given rotation $\alpha_{\mathrm{WP}}$:

$$J_{\mathrm{rot}}(\alpha_{\mathrm{WP}})=(\begin{array}{cc} \cos\left(\alpha_{\mathrm{WP}}\right) & \sin\left(\alpha_{\mathrm{WP}}\right)\\ -\sin\left(\alpha_{\mathrm{WP}}\right) & \cos\left(\alpha_{\mathrm{WP}}\right) \end{array}).$$ (4)

The Jones matrix of a wave plate is given by:^43^^,^^45^^,^^46^

$$J_{\mathrm{WP}}(\Delta \phi )=(\begin{array}{cc} e^{i\frac{\Delta \phi }{2}} & 0\\ 0 & e^{-i\frac{\Delta \phi }{2}} \end{array}).$$ (5)

The retardation Δϕ of the wave plate depends on the current wavelength, the refractive indices of the used birefringence material of the ordinary $n_{o}$ and extraordinary $n_{e}$ ray, and the thickness $d_{\mathrm{WP}}$ of the element. The retardation can be calculated by^43^^,^^45^

$$\Delta \phi (\lambda )=\frac{2\pi }{\lambda }d_{\mathrm{WP}}(n_{e}-n_{o}).$$ (6)

The difference in the quartz refractive index of the used spectral range can be computed, using the three-term form of the empirical Sellmeier equation and the given coefficients for the refractive index of quartz crystal.^47^

$$n_{o,e}^{2}=A_{o,e}+\frac{B_{o,e}\lambda^{2}}{\lambda^{2}-C_{o,e}}+\frac{F_{o,e}\lambda^{2}}{\lambda^{2}-G_{o,e}}.$$ (7)

By inserting Eq. (5) in Eq. (3) and some mathematical transformations, the final Jones matrix for a rotated retarder is obtained.^45^

$$J_{\mathrm{WP},\mathrm{rot}}(\lambda ,\alpha_{\mathrm{WP}})=(\begin{array}{cc} e^{i\frac{\Delta \phi \left(\lambda \right)}{2}}\;\cos^{2}\;\alpha_{\mathrm{WP}}+e^{-i\frac{\Delta \phi \left(\lambda \right)}{2}}\;\sin^{2}\;\alpha_{\mathrm{WP}} & (e^{i\frac{\Delta \phi \left(\lambda \right)}{2}}-e^{-i\frac{\Delta \phi \left(\lambda \right)}{2}})\cos\;\alpha_{\mathrm{WP}}\;\sin\;\alpha_{\mathrm{WP}}\\ (e^{i\frac{\Delta \phi \left(\lambda \right)}{2}}-e^{-i\frac{\Delta \phi \left(\lambda \right)}{2}})\cos\;\alpha_{\mathrm{WP}}\;\sin\;\alpha_{\mathrm{WP}} & e^{i\frac{\Delta \phi \left(\lambda \right)}{2}}\;\sin^{2}\;\alpha_{\mathrm{WP}}+e^{-i\frac{\Delta \phi \left(\lambda \right)}{2}}\;\cos^{2}\;\alpha_{\mathrm{WP}} \end{array}).$$ (8)

By computing the intensity for each wavelength of the used spectral range using the Jones vector of the system and its complex conjugate [see Eq. (9)],^44^^,^^45^ the resulting intensity distribution can be interpreted as transmission spectrum $T_{\mathrm{PF}}$ of the polarization-based spectral filter.

$$T_{\mathrm{PF}}(\lambda )=J_{\text{out}}^{*}(\lambda )J_{\text{out}}(\lambda ).$$ (9)

To compute the measured intensity at the image sensor, the spectra of the overall system has to be considered. This simulated final spectrum at the camera sensor is a product of the tissue spectra (ROI dataset), the transmission or sensitivity of the optical components, and the transmission spectrum of the polarization spectral filter system $T_{\mathrm{PF}}$. The camera sensor now integrates the detected spectra over the used spectral range, resulting in an intensity of each individual spectral channel (four intensity values), encoding the previous tissue spectrum.

To optimize the classification results, this is done for various wave plates and rotations, leading to a dataset of encoded tissue spectra and the used wave plates and rotations. A LightGBM algorithm and a NN are used for classification, which not only take the four intensity values into account but also the normalization of these four intensity values.

The NN uses three hidden layers, with the first layer consisting of 12 nodes, which are halved 2 times. The activation function is again a ReLU function. The output layer results in a binary result, computed by the sigmoid function. The loss during training is calculated by binary crossentropy.

Tables 7 and 8 show the classification results of the used setup for simulated data. As can be seen, the polarization-based spectral filtering performs comparably good to the results obtained by CS or PCA (compare Tables 5 and 6).

**Table 7:** Classification results of the ROI test datasets for healthy and malignant tissue with the polarization spectral filtering.

Dataset	Algorithm	Accuracy (%)	Precision (%)	Recall (%)	F1-Score (%)
ROI	NN	70	77	70	71
ROI	LightGBM	74	79	74	74
Image	NN	77	79	77	78
Image	LightGBM	79	80	79	79

**Table 8:** Classification results of the ROI test datasets for subclasses of healthy and malignant tissue with the polarization spectral filtering.

Dataset	Algorithm	Accuracy (%)	Precision (%)	Recall (%)	F1-Score (%)
ROI	NN	62	63	62	61
ROI	LightGBM	63	62	63	61
Image	NN	46	49	46	43
Image	LightGBM	47	48	47	44

## 3 Results

### 3.1 Illumination-Based Approach

To characterize the system, the spatial resolution, the field of view as well as the intensity of each spectral channel is considered. The field of view is evaluated using a chessboard target with 3 mm square size. The working distance is set to ∼10  mm resulting into a lateral object width and height of ∼20  mm and therefore a field of view of 90 deg. This confirms that the field of view is not changed to the field of view of the commercial endoscope.

**Fig. 7:**
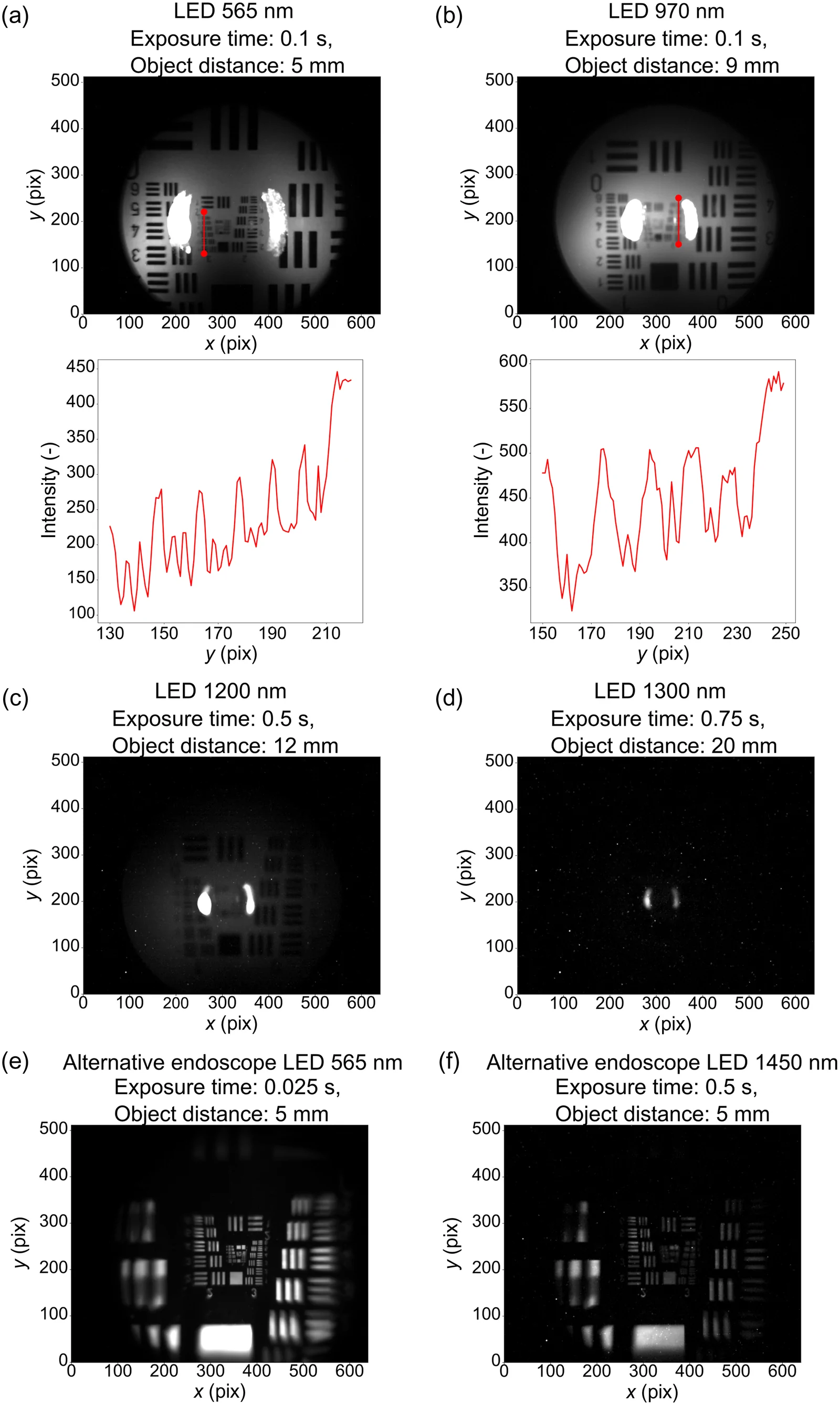
Measurement of the USAF target by the endoscopic system using the light source for illumination. It can be seen that the image intensity decreased with increasing wavelength. The varying object distance indicates that chromatic aberrations occur. By using the endoscope mimicking setup (e) and (f), a comparable resolution can be achieved for the smallest and largest wavelength. In addition, the image intensity can be adapted within the exposure time. The reflection of the endoscopes distal illumination path can be seen in panels (a)–(d).

Spatial resolution was measured by a USAF target. The results are shown in Fig. 7, according to the used object distance and used exposure time per spectral channel. It can be clearly seen that there is a large reduction in image intensity by increasing the illumination wavelength. In addition, it seems that chromatic aberrations occur, which lead to the need of readjustment of the object distance for each spectral channel. These chromatic aberrations as well as the reduction of image intensity are mainly induced by the endoscope, as is validated by a customized setup using a VIS-SWIR objective lens (Kowa LM50HC-VIS-SW) to mimic the endoscope. The light source is used for illumination and the imaging is done by the proposed imaging system (bottom Fig. 7).

The signal-to-noise-ratio (SNR) of the endoscopic sensor system is determined by measuring a spectralon surface (Sphere Optics, Zenith Polymer Reflectance Standard). For this, an image sequence of 100 images has been taken and evaluated for a single pixel over 100 images (temporal), a square ROI (edge length 24 pixels) in the center of the image for one image (spatial), and the ROI over 100 images (combined). The results can be seen in Table 9, showing that especially the SWIR channels currently do not allow for imaging with desired quality.

**Table 9:** Signal-to-noise-ratio by measuring a spectralon. The decreased spatial SNR values indicate an inhomogenous illumination by the endoscope illumination path.

LED	Temporal	Spatial	Combined
565	203	35	35
970	301	54	54
1200	102	20	20
1300	45	10	9
1450	26	2	2

To validate the loss in image intensity, the irradiance of the system is roughly measured by two calibrated spectrometers (Avantes AvaSpec-ULS2048CL-EVO and AvaSpec-NIR256-1.7-EVO), showing, that especially the imaging path of the endoscope blocks light of wavelengths>1000  nm. This leads to the need of an optimized endoscopic system to allow for imaging in the SWIR spectral range as well. With respect to the needed exposure times, real-time-capable image acquisition can until then only be achieved for two of the given CS spectral channels. The 565 nm channel needed 100 ms in case of the endoscopic setup with reduced irradiance of the LED and 25 ms in case of full irradiance with the alternative setup. The 970 nm channel needed 100 ms in case of endoscopic and 25 ms in case of alternative setup.

**Fig. 8:**
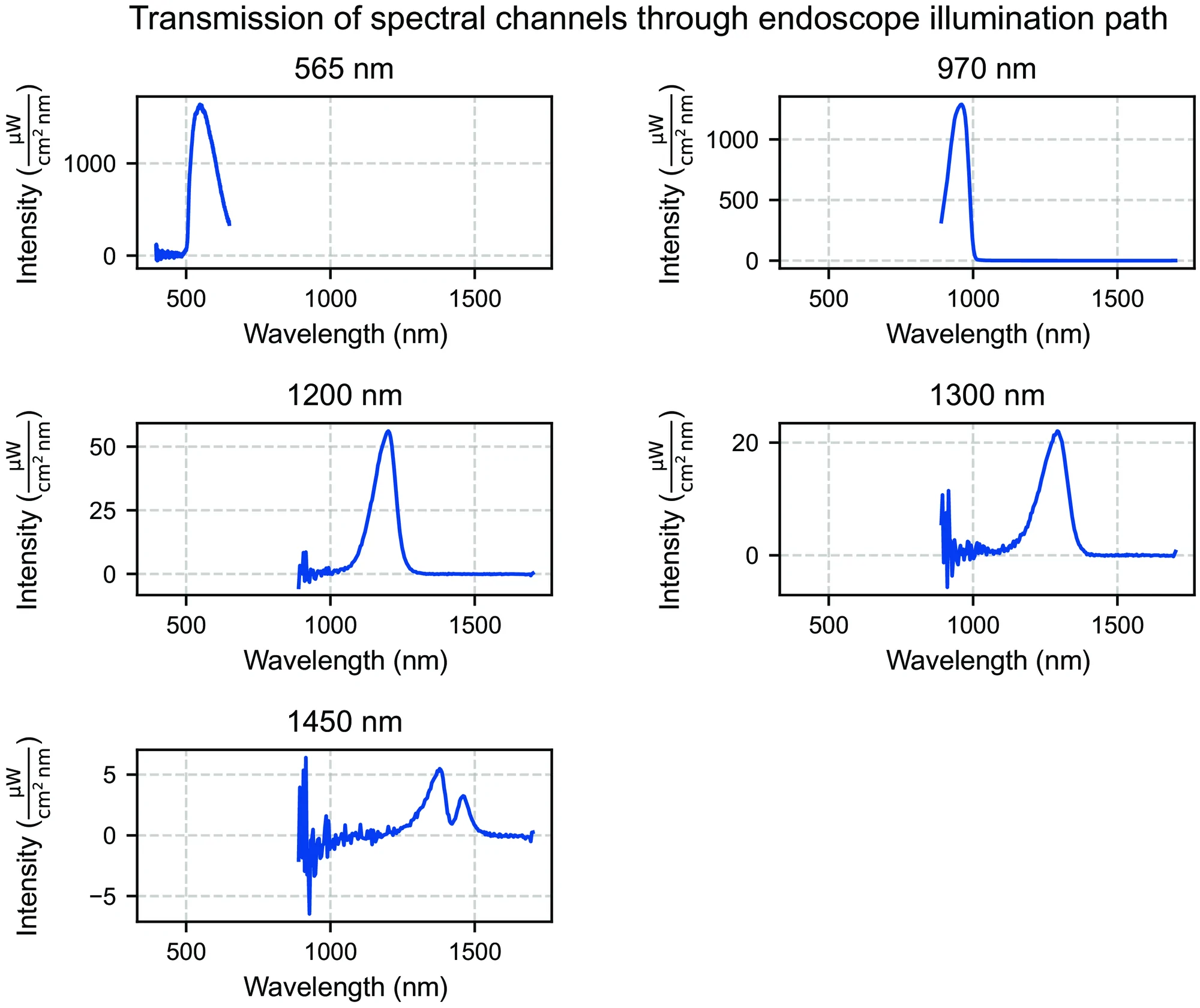
Spectra of the spectral channels in the object plane after passing the light guide and the illumination path of the endoscope. All wavelengths>1000  nm are damped by the endoscope. It can be seen that especially the 1450 nm LED is influenced by the light guide of the endoscope.

The spectral output at the distal end of the endoscope is shown in Fig. 8. All LEDs are set to their maximum, indicating, that in case of combining LEDs to common illumination modes, as for PCA spectra, the intensity of each LED has to be adapted, to use a homogenous weighting of the single spectral ranges. For the 1450 nm LED, a reduction around 1400 to 1450 nm can be seen, which is not present by measuring the LEDs spectrum directly at the LED. The spectrum is thus altered by the endoscopes illumination path and can be attributed to the light guide and may be induced by the OH absorption of the glass material.

Because the transmission in the SWIR is limited, imaging is only reasonable until now for a spectral range below 1000 nm. This leads to a reduction to only two spectral channels in case of spectra obtained by CS. In case of using the spectra obtained by PCA (or in case of polarization-based spectra using the imaging-based sensor) still four channels remain, but are also limited. Therefore, the final validation of both sensor modalities will be postponed until sufficient transmission in the SWIR range can be achieved.

**Fig. 9:**
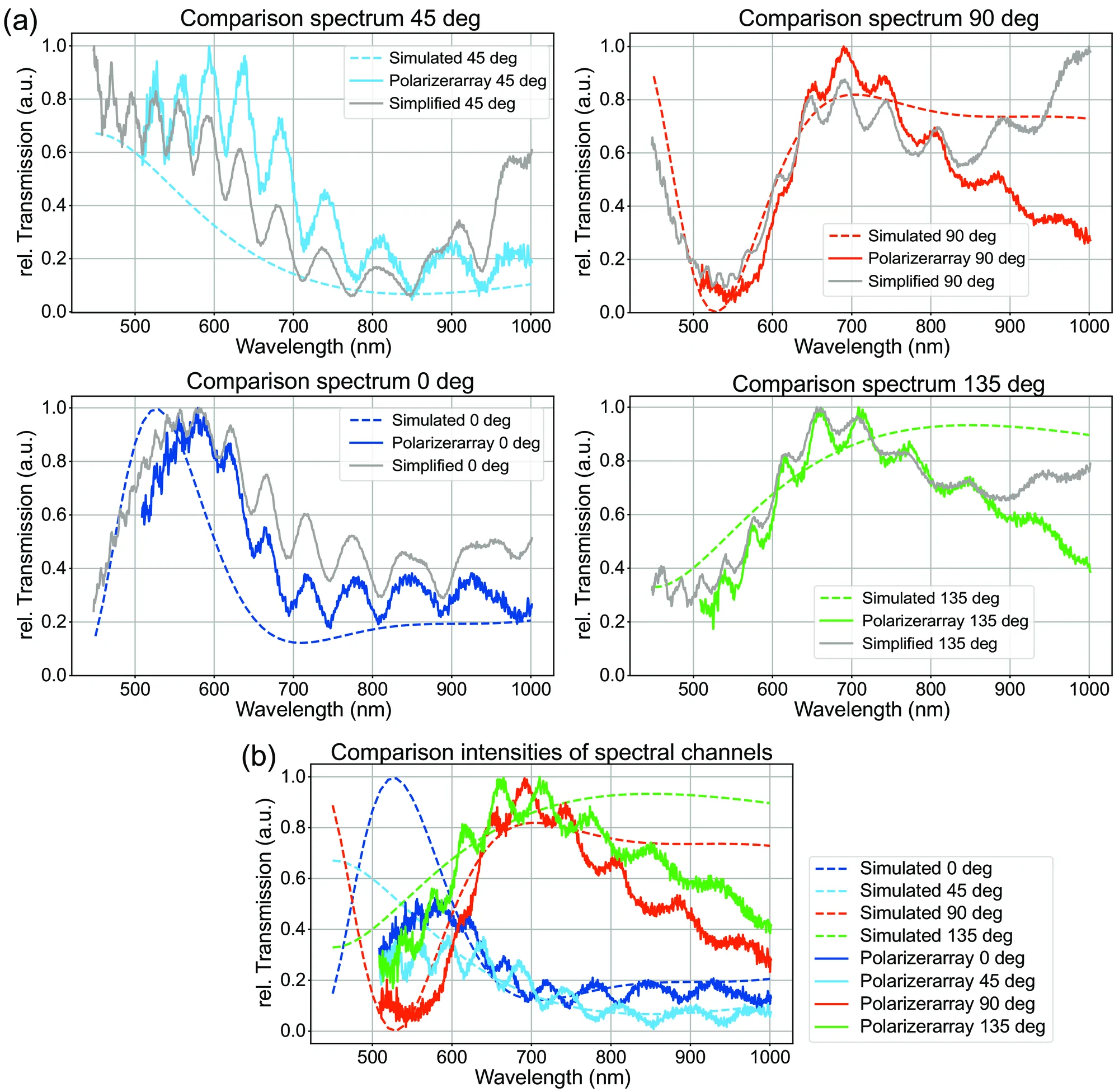
Comparison of simulated (colored dashed) and measured spectra per spectral channel using the polarizer array (colored) or the simplified setup (gray) (a). The different intensities (normed to the overall maximum) can be seen in (b). Intensity fluctuations between the spectral channels might be caused by repositioning the spectrometer to measure the individual apertures of the polarization filter array as well as in reduced extinction ratio and transmission of the polarizer due to the joining procedure. The superposed oscillation might be attributed to reflections and interference effects between the waveplates, input polarizer and long-pass filter.

To quantify the degradation in classification ability, the LightGBM classifier is trained and evaluated with 1000 nm as upper spectral limit. The results are shown in Table 10. As can be seen, the results reduce (compared to the maximum possible score of 100%) in the range of 1% to 2% in case of PCA spectra, 2% to 5% for CS spectra and 2% to 5% for polarization-based spectral filtering (imaging-based sensor system). As expected, the spectra by PCA show the lowest reduction, as the spectral channels still cover several important spectral regions and show variance within the four spectral channels spectra. In case of CS the reduction also is expectable, as only two channels remain. The reduction of the polarization-based spectra, show that even using complex spectra for this sensor system, the information is however, limited to the VIS region, as the four channels spectra show comparable behavior between 650 and 1000 nm for 0 deg and 45 deg and for 90 deg, and 135 deg [see Fig. 9(b)], leading to almost no additional information above 650 nm is added. It can therefore be assumed, that increasing the spectral range to the SWIR is however, beneficial, as more (biological interpretable) information is usable for the classification task and therefore can be used for plausibility issues.

**Table 10:** Classification results (LightGBM) of the ROI and images datasets for main tissue classes and reduced spectral information with 1000 nm as upper spectral limit.

Dataset	Spectral channels	Accuracy (%)	Precision (%)	Recall (%)	F1-Score (%)
ROI	CS	74	80	74	74
ROI	PCA	75	81	75	75
ROI	Polarization	69	78	69	70
Image	CS	79	79	79	79
Image	PCA	80	80	80	80
Image	Polarization	77	76	77	76

Besides the adapted SWIR capable endoscope, further optimization potential of the illumination-based sensor system lies in the coupling and triggering of the LEDs, the image acquisition, as well as in implementing an optimized image processing pipeline to allow for real-time data analysis.

### 3.2 Imaging-Based Approach

According to the illumination-based approach, the polarization-based spectral filtering sensor is also validated with respect to its spectral behavior, the field of view and the spatial resolution. As the endoscope blocks light >1000  nm, the spectral characteristics are only compared in the range between 450 and 1000 nm.

Figure 9 shows the transmission spectra of each spectral channel in comparison to the simulated spectra (colored dashed lines). In addition to the polarization array (colored lines), a second simplified setup was used to quantify the spectra (grey lines), only consisting of the input polarizer, the long-pass filter, the two waveplates and a rotatable analyzer. The spectra are measured by a spectrometer (AvaSpec-ULS2048CL-EVO). As can be seen, the spectra fit more or less the simulated spectra. Deviations might be attributed due to adjustment errors in rotation and tilt of the polarization elements. In addition, the joining process causes deformations in the polarization array. Because these are polymer-based polarizers, this immediately leads to changes in the polarization states due to birefringence, resulting in deviations in the measured spectra. Furthermore, the extinction ratio as well as the transmission of the film polarizers is probably reduced at the edges due to the cutting process.

The superposed oscillation, visible in the spectra of the polarization array as well as for the simplified setup, might be attributed to internal reflections and interference effects between the wave plates, the input polarizer and the long-pass filter, because these components are mounted in a common holder, which was used for both measurements.

**Fig. 10:**
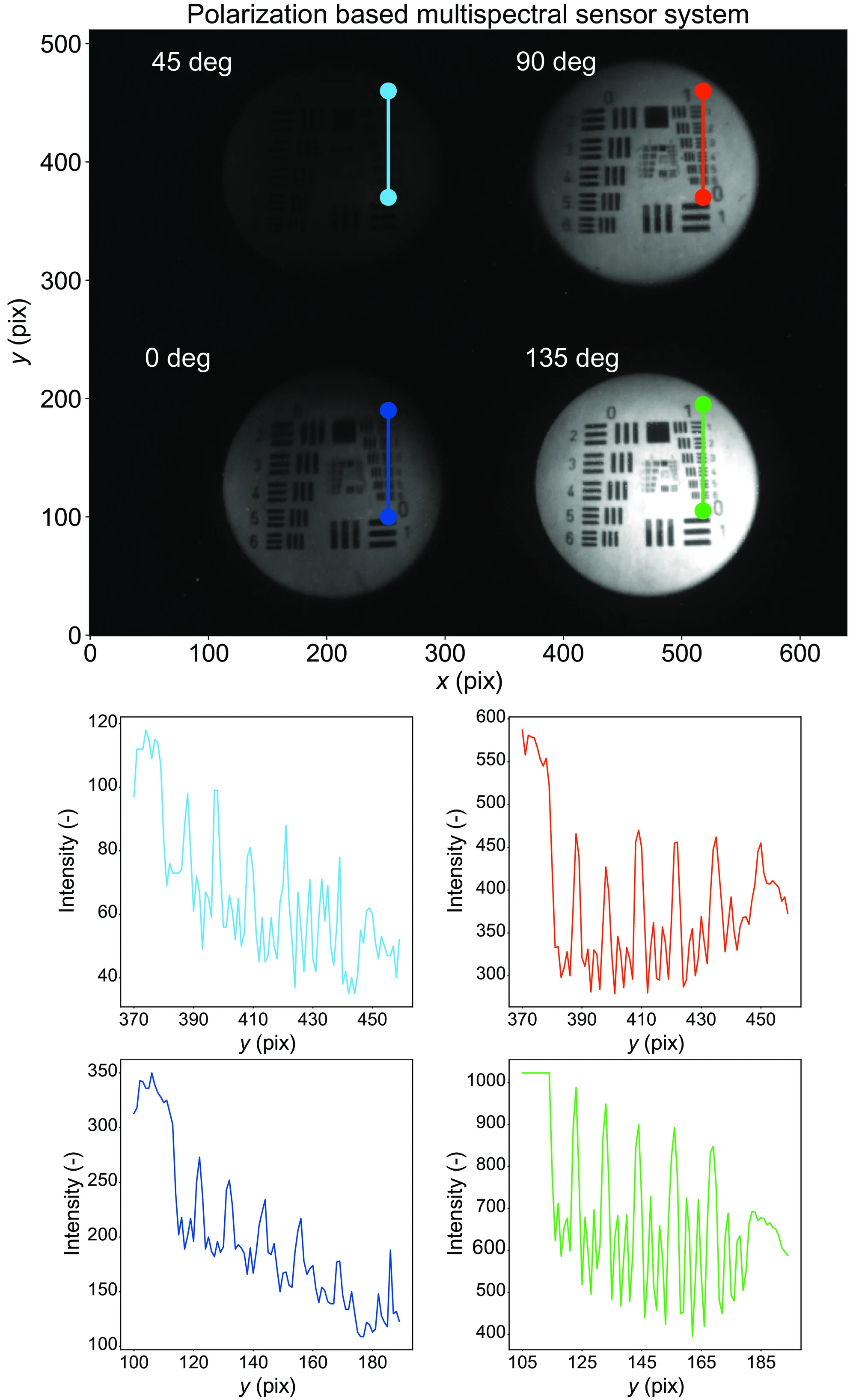
Measurement of the USAF target with the polarization-based sensor system. The differences in image intensity can be seen, especially for the 0 deg and 45 deg channels. By an object distance of 10 mm, a spatial resolution of 140  μm can be measured.

In addition, each spectral channel is expected to show different image intensities (by integrating over the single transmission spectra), which was confirmed by measuring the USAF target, see Fig. 10. It can be seen, that especially the 0 deg and 45 deg channels appear comparably dark, which might additionally be caused by a 10% to 20% reduced quantum efficiency of the image sensor in that spectral range and the spectral characteristic of the used halogen light source (Thorlabs OSL2IR). Nevertheless, an object size of 140  μm (element 1.6) with an exposure time of 80 ms can be measured by all channels. For this, the positioning of the lenses of the relay lens setup has to be adjusted, which might be attributed to a too large distance between the lens array and the image sensor because of the camera coverglass.

The used working distance was set to 10 mm, yielding in an object field of around 20 mm and hence 90 deg field of view. The SNR is obtained by measuring the spectralon with an exposure time of 15 ms. The results are listed in Table 11, showing a reduced SNR for the two darker spectral channels.

**Table 11:** Signal-to-noise-ratio by measuring the spectralon with halogen white light illumination coupled to the endoscope illumination path. As can be seen by decreased spatial SNR, the illumination is inhomogenous due to the endoscope’s distal end (also can be seen in Fig. 7 by the reflection on the USAF target). In addition, the spectral channels for 0° and 45° show a decreased image intensity.

Channel	Temporal	Spatial	Combined
0°	84	32	32
45°	34	14	14
90°	116	39	39
135°	166	36	35

## 4 Discussion

The presented study shows that tissue differentiation of healthy and malignant breast tissue is also possible with only few spectral channels in the visible to shortwave infrared spectral range. To define the spectral channels we used a method based on curve sketching, PCA and LDA. All methods show similar spectral ranges and therefore confirm the importance of these wavelengths. Similarly the spectra of the system, based on polarization dependent spectral filtering, show the same behavior, by weighting the ranges around 450 to 600 nm (0 deg, 45 deg, and 135 deg), but also the range above 1200 nm (0 deg and 135 deg). The range between 800 and 1100 nm is represented by the 90 deg and 135 deg channels [see Fig. 6(b)]. The importance of these regions become apparent, by comparing them to tissue chromophores such as lipids, water, collagen, and β-carotene, representing main tissue components of healthy and malignant breast tissue.

Classifications with reduced spectral information still show good results, especially for the two class problem. In case of the subclass problem, the results are reduced due to imbalance of the dataset, the small sample size per subclass and due to a higher similarity between the single subclasses (e.g., ’adipose’ and ’adipose and stroma’). In addition, precursor lesions show only minor impact to the surrounding healthy tissue and are therefore more complex to detect, as it is assumed, that tumor regions are primarily detected indirectly by their impact to the healthy tissue (tumor microenvironment). Reducing the number of spectral channels therefore affects the classification algorithms ability to compensate for uncertainties or only slight differences in the tissue spectra. A possible solution might be to increase the number of reduced spectral channels, to allow for more information being used. Because the use of subclasses was recommended by the pathology department after sensor development had already begun, it was no longer possible to modify the spectral channels at the hardware level at that point. Given the comparable results of the spectral analysis, the same number of spectral channels was used for the classification of the subclasses, with only minor variations in their spectra as mentioned above. In general, both miniaturized systems allow for integration of additional spectral channels. However, the increase in measurement time (illumination-based approach) or the loss in spatial resolution (imaging-based approach) has to be considered. Another option is to increase the spectral channels complexity. With CS spectra, only individual bands are used for each channel, whereas PCA represents more complex patterns and therefore achieves better classification results. Hence, a subsequent optimization of the spectra of each spectral channel might increase the ability for tissue differentiation in case of main- and subclass analysis.

Generally, the amount of patients contributing to this study is still low. To gain more robust spectral characteristics and classification, further measurements have to be carried out. The attention has to lie on a diverse variety of pathological diagnoses, so that a balanced dataset can be achieved. In addition, trying to reduce the average age of the patients is another challenge, as it is to be expected that younger patients show different tissue composition in healthy tissue as older ones.

Because the training and test dataset show a differing tissue type distribution in the context of imbalanced dataset and small sample size, applying methods for cross validation or homogenization of the used classes might improve the classification. In addition, it might demonstrate dependencies resulting from the datasets imbalance, which were included here.

For further analysis with regard to tumor margin assessment, taking the per-class sensitivity (recall) and specificity into account is necessary. This might also illustrate the real differentiation ability in case of subclasses, which was only presented by a weighted mean value in this study. However, for this analysis, it is also advisable to include a larger, as balanced as possible, number of patients. In addition, the registration process has to be optimized for this task as well, as there are uncertainties now, which might influence the more distinct per-class evaluation at this point.

To overcome misclassifications, optimization of the used basic algorithms might be advantageous. Further potential could lie in the adaption of the classification strategy. Instead of classifying the tissue classes directly a stepwise differentiation process might be beneficial, which first uses a general tissue type differentiation in healthy and tumor and subsequently verifies the tumor class with a more specific analysis for the tumor subclasses (tumor and precursor) and the healthy stroma tissue. Another main optimization can be achieved by taking the spatial information into account. Until now, only spectral differences were analyzed, neglecting valuable information given by the image data. Additional plausibility checks by image processing and logical operations might also reduce misclassifications, as single falsely classified pixels in large areas of the other tissue class, might be attributed to noise, dead pixels or specular reflections.

Another great potential lies in the evaluation of the probability maps, as shown in Rüdinger et al.^33^ Because the results mentioned above only show the outcomes of majority voting, incorporating probability can help to highlight uncertainties in the tissue transition, as in tumor margins. By offering the reached probability, the surgeon can either intervene or select a decision strategy, so that the prediction, and hence the tissue removal, is made more conservative or strict.

Given the prediction probability and the uncertainties associated with tissue transitions, the overall prediction can also be improved by validating the results using other sensor modalities and subsequent sensor fusion.^48^ Sensor fusion might especially be beneficial in cases of subclasses or a combined main- and subclass evaluation, to first evaluate the rough estimate of the tumor margins and subsequently, evaluate the tissue transition or plausibilze the tissue type. A promising sensor modality is the usage of elastography, as was tested in Rüdinger et al.,^31^ as different types of healthy tissue also have different haptic feedback. In addition, electrical impedance measurement,^49^ by differing electrical properties as conductivity, or the evaluation of optical scattering and absorption properties, might be advantageous.

With the spectral channels described in this work, two sensor systems, an illumination and an imaging-based approach have been presented. Both modalities show good spatial resolution and representation of the defined spectral channels. However, the transmission of the endoscope blocks light >1000  nm, which prevents the use of SWIR information and thus partially or completely limits the usability of the individual spectral channels. Because fused silica is generally transparent across a broad spectral range (VIS to IR), antireflective coatings could limit transmission. Optimizing the endoscope system is therefore a further necessary step in validating the sensor systems. Because no endoscopes are currently available for such broad spectral ranges, using an older endoscope system with less stringent coating requirements could be a possible solution.^50^ Another option is to develop an endoscope prototype that enables transmission in the SWIR range. Nevertheless, this option is challenging, as the optics need to be optimized for the entire spectral range to enable for full field of view imaging at a comparable working distance across all wavelength bands. Alternatively, as a medium-term solution, an imaging fiber bundle placed in the endoscope’s working channel could be used for the imaging path. However, this option would have the disadvantage that the fiber might have to be removed from the working channel to use the surgical instruments, meaning that no measurement would be available at the crucial moment.

The polarization-based setup can further be miniaturized, if polarization image sensors, already commerically available for the VIS-NIR spectral range, will be expanded to SWIR range as well. If so, an equivalentely small setup as for the shown imaging system of the illumination-based approach will be possible.

Because the images of the illumination-based approach are captured sequentially, the alignment of the single spectral images might be challenging. Tracking and stitching methods as proposed by Yoon et al.^51^ and von Braun et al.^52^ for HSI linescan setups or identification of characteristic landmarks, as for endoscopic bladder procedures in Krauß et al.,^53^ might be useful for this setup in an adapted way. By tracking object key-points during freehand endoscopic measurement, the single spectral images can be aligned, to evaluate the full multispectral image cube.

## 5 Conclusion

We presented two miniaturzied measurement systems, which perspectively allow for intraoperative endoscopic tissue differentiation. For this purpose, we used few but meaningful spectral channels, derived by spectral analysis. By reducing the amount of spectral channels to only four channels, the image acquisition can be accelerated and enables potential real-time-capable measurement. An endoscope, which also allows for transmission in the SWIR spectral range, enables the full usage of all spectral channels, defined in this work and allows further (biological interpretable) information to be used for classification. With further optimization of the data processing, a real-time data evaluation and tissue type prediction can be realized, enabling real-time, intraoperative use of multispectral endoscopic systems for tissue differentiation.

## Data Availability

All data are available from the corresponding authors upon reasonable request.
